# relA over-expression reduces tumorigenicity and activates apoptosis in human cancer cells

**DOI:** 10.1054/bjoc.2001.2174

**Published:** 2001-12

**Authors:** A Ricca, A Biroccio, D Trisciuoglio, M Cippitelli, G Zupi, D Del Bufalo

**Affiliations:** Experimental Chemotherapy Laboratory, Laboratory of Pathophysiology, Regina Elena Cancer Institute, Via delle Messi d'Oro 156, Rome, 00158, Italy; Department of Experimental Medicine and Pathology, La Sapienza University of Rome, Piazzale Aldo Moro 5, Rome, 00185, Italy

**Keywords:** relA, breast carcinoma, tumorigenicity, apoptosis

## Abstract

We previously demonstrated that bcl-2 over-expression increases the malignant behaviour of the MCF7 ADR human breast cancer cell line and enhances nuclear factor-*kappa* B (NF-*k* B) transcriptional activity. Here, we investigated the direct effect of increased NF-*k* B activity on the tumorigenicity of MCF7 ADR cells by over-expressing the NF-*k* B subunit relA/p65. Surprisingly, our results demonstrated that over-expression of relA determines a considerable reduction of the tumorigenic ability in nude mice as indicated by the tumour take and the median time of tumour appearance. *In vitro* studies also evidenced a reduced cell proliferation and the activation of the apoptotic programme after relA over-expression. Apoptosis was associated with the production of reactive oxygen species, and the cleavage of the specific substrate Poly-ADP-ribose-polymerrase. Our data indicate that there is no general role for NF-*k* B in the regulation of apoptosis and tumorigenicity. In fact, even though inhibiting NF-*k* B activity has been reported to be lethal to tumour cells, our findings clearly suggest that an over-induction of nuclear NF-*k* B activity may produce the same effect. © 2001 Cancer Research Campaign http://www.bjcancer.com


*British Journal of Cancer* (2001) **85**(12), 1914–1921
© 2001 Cancer Research Campaign
doi: 10.1054/ bjoc.2001.2174, available online at http://www.idealibrary.com on IDEAL

http://www.bjcancer.com

### relA over-expression reduces tumorigenicity and activates apoptosis in human cancer cells

**A Ricca[1], A Biroccio[1], D Trisciuoglio[1], M Cippitelli[2,3], G Zupi[1] and D Del Bufalo[1]**

[1]Experimental Chemotherapy Laboratory and [2]Laboratory of Pathophysiology, Regina Elena Cancer Institute, Via delle Messi d'Oro 156, 00158 Rome, Italy;
[3]Department of Experimental Medicine and Pathology, La Sapienza University of Rome, Piazzale Aldo Moro 5, 00185, Rome, Italy

**Summary** We previously demonstrated that bcl-2 over-expression increases the malignant behaviour of the MCF7 ADR human breast cancer cell line and enhances nuclear factor-*kappa* B (NF-*k*B) transcriptional activity. Here, we investigated the direct effect of increased NF-*k*B activity on the tumorigenicity of MCF7 ADR cells by over-expressing the NF-*k*B subunit relA/p65. Surprisingly, our results demonstrated that over-expression of relA determines a considerable reduction of the tumorigenic ability in nude mice as indicated by the tumour take and the median time of tumour appearance. *In vitro* studies also evidenced a reduced cell proliferation and the activation of the apoptotic programme after relA over-expression. Apoptosis was associated with the production of reactive oxygen species, and the cleavage of the specific substrate Poly-ADP-ribose-polymerrase. Our data indicate that there is no general role for NF-*k*B in the regulation of apoptosis and tumorigenicity. In fact, even though inhibiting NF-*k*B activity has been reported to be lethal to tumour cells, our findings clearly suggest that an over-induction of nuclear NF-*k*B activity may produce the same effect. © 2001 Cancer Research Campaign http://www.bjcancer.com

**Keywords**: relA; breast carcinoma; tumorigenicity; apoptosis

The mammalian nuclear factor-*kappa* B (NF-*k*B) family comprises 5 members (p50, p52, relA/p65, c-rel and relB) which form a different array of homo-and hetero-dimers and are regulated by a group of citoplasmic inhibitors (IκBs) (Krappmann et al, 1999). In unstimulated cells, it is retained in an inactive cytoplasmatic form by one of several IκB molecules. Activation of NF-*k*B complexes by a variety of stimuli results in the phosphorylation and rapid degradation of IκBs through the ubiquitin-proteasome pathway (Thanos and Maniatis, 1999). Dissociation of IκB from NF-*k*B allows NF-*k*B to translocate to the nucleus and bind to κB DNA sites with the consequent activation of κB-target gene expression (Baeuerle and Henkel, 1994).

The rel/NF-*k*B/IκB superfamily of signal transducers and transcription factors exists in virtually all cell types but has been characterized as a mediator of response to several stimuli in the immune system (Baeuerle and Henkel, 1994; Krappmann et al, 1999; Thanos and Maniatis, 1999). More recently, it has been shown that NF-*k*B can also regulate diverse cellular processes such as apoptosis (Abbadie et al, 1993; Lin et al, 1995; Carter et al, 1996; Grilli et al, 1996; Grimm et al, 1996; Wang et al, 1998; Wu et al, 1998; Pahl, 1999; Lin et al, 1999; Grilli and Memo, 1999; Kaltschmidt et al, 2000), cell cycle (Baeuerle and Baltimore, 1996; Bargou et al, 1997; Bash et al, 1997; Seitz et al, 1998; Sheehy and Schlissel, 1999) and oncogenesis (Higgins et al, 1993; Gilmore, 1997; Nakshatri et al, 1997; Sovak et al, 1997; Visconti et al, 1997; Rayet and Gélinas, 1999; Andela et al, 2000; Huang et al, 2000).

A variety of studies encompassing a broad range of both cell types and apoptosis-inducing stimuli have evidenced that activation of NF-*k*B plays a role in both protecting and inducing apoptosis. In a number of systems, NF-*k*B has been demonstrated to have an anti-apoptotic function, and several NF-*k*B responsive anti-apoptotic genes (*clAP1, clAP2, TRAF1, TRAF2, IEXL-1*) have recently been identified and claimed to play a role in this process (Wang et al, 1998; Wu et al, 1998; Pahl, 1999). Activation of NF-*k*B has also been correlated with the activation of the apoptotic programme in a wide variety of systems such as avian embryonic development, ceramide-activated osteoblasts, dopaminergic neurons derived from Parkinson disease patients, bone marrow cells and prostate carcinoma cells (Abbadie et al, 1993; Lin et al, 1995,1999; Carter et al, 1996; Grilli et al, 1996; Grimm et al, 1996). The pro- and anti-apoptotic regulatory function of NF-*k*B has been shown to depend on the cell type, the differentiation state of the cell, and the nature of the apoptotic stimulus (Abbadie et al, 1993; Lin et al, 1999; Kaltschmidt et al, 2000). Given such a divergent outcome, it has been suggested that NF-*k*B activation acts as a checkpoint between cell rescue and apoptosis (Grilli and Memo, 1999).

As reported for the NF-*k*B role on apoptosis, a different effect on cell proliferation has also been demonstrated. In fact, in contrast to its role in HeLa cells and in a transformed pro-B cell line where NF-*k*B leads to growth arrest, and in transgenic epithelium where it produces hypoplasia, NF-*k*B proteins promote cellular proliferation in malignant lymphoma (Baeuerle and Baltimore, 1996; Bargou et al, 1997; Bash et al, 1997; Seitz et al, 1998; Sheehy and Schlissel, 1999).

Thus, the cell type and the context of a NF-*k*B-inducing stimulus appear to be crucial in determining the outcome of a signal that can lead to proliferation, differentiation or cell death.

Persistent nuclear NF-*k*B activity has been described in several human cancer cell types, where chromosomal amplification, over-expression and rearrangement of genes coding for *rel*/NF-*k*B factors have also been outlined. A continuous activation of NF-*k*B factor is emerging as an indicator of various types of solid tumours

*Received 17 April 2001*
*Revised 24 August 2001*
*Accepted 25 September 2001*

*Correspondence to:* D Del Bufalo

(Gilmore, 1997) and as an important factor in the onset and progression of several human tumours including breast carcinoma (Bargou et al, 1997; Visconti et al, 1997; Rayet and Gélinas, 1999; Huang et al, 2000). Breast cancer cell lines, breast cancer specimens and a majority of carcinogen-induced mammary tumours in rats exhibit high levels of nuclear NF-*k*B DNA-binding activity, and a link has been suggested between constitutive activation of NF-*k*B and tumour progression to the more aggressive anti-oestrogen-resistant phenotype and in general with the acquisition of invasive-metastatic properties (Higgins et al, 1993; Nakshatri et al, 1997; Sovak et al, 1997; Andela et al, 2000).

However, little is known about how constitutive NF-*k*B activity contributes to the malignancy of cancer cells. Several studies, demonstrating that NF-*k*B activity contributes to cell survival and abnormal cell proliferation of tumour cells, seem to indicate that rel transcription factor may provide accessory functions for cancer development.

We have previously shown that the increase of tumorigenic, metastatic and angiogenic potential of the MCF7 ADR human breast cancer cell line in consequence of bcl-2 over-expression is associated with an enhanced NF-*k*B-mediated transcriptional activity (Del Bufalo et al, 1997; Biroccio et al, 2000; Ricca et al, 2000). Here, we investigated the direct role of NF-*k*B sustained over-induction on the tumorigenesis of the MCF7 ADR cell line. These cells were transfected with an expression vector for *relA* and 3 stable clones over-expressing relA were utilized for *in vitro* studies and evaluation of tumorigenic potential in nude mice. We found that the constitutive relA over-expression in MCF7 ADR cells results in inhibition of tumorigenic ability, associated with reduced cell proliferation and activation of the apoptotic programme.

### MATERIAL AND METHODS

### Cell lines and culture conditions

The MCF7 ADR human breast cancer, the M14 melanoma and the H460 lung adenocarcinoma lines were maintained in RPMI 1640 supplemented medium (Gibco, Gaithersburg, MD, USA). MCF7 ADR cells were cultured in 10 μM Adriamycin (ADR), and grown for 2 weeks in drug-free medium prior to each experiment. Cell growth was assessed by seeding $2 \times 10^5$ cells in 60-mm plates (Nunc, Mascia Brunelli, Milan, Italy). Cell counts (Coulter Counter, Kontron Instruments, Milan, Italy) and viability (trypan blue dye exclusion) were determined daily, from day 1 to day 8 of culture.

### Transfection

Cells (~$1 \times 10^6$ cells in 250 μl medium) were transfected by electroporation (280 V, 950 μF, Gene Pulser, Bio-Rad, Milan, Italy) with the expression vector pcDNA3 (Invitrogen BV, NV Leek, The Netherlands) carrying the *relA* cDNA and the gene for resistance to Neomicine (*neo*, Gibco). As control, cells were transfected with the plasmid carrying the *neo*-resistance gene alone. Transfected clones were collected 20 days after selection in neo-containing medium (800 μg ml$^{-1}$) and relA expression was tested by Western blotting on nuclear extracts obtained as previously reported (Ricca et al, 2000).

### Western blotting

To evaluate the expression of relA, p53, bcl-2, bax, bcl-$x_{L/S}$, c-myc and Fas-Ligand (Fas-L) proteins or the cleavage of Poly-ADP-ribose-polymerrase (PARP) substrate total cells or nuclei were incubated at 4˚C for 30 min in lysis buffer with ionic detergent (2% SDS; 20 mM Tris pH 8.0; 2 mM PMSF). 40 μg of nuclear extracts or total proteins from each sample were separated by SDS-PAGE. Western blot and detection were performed as previously reported (Del Bufalo et al, 1996). Antibodies specific for human p65 (C-20; Santa Cruz Biotechnology, Santa Cruz, CA, USA; dil. 1:5000), p53 (clone Pab 1801; Santa Cruz Biotechnology; dil. 1:500), c-myc (clone 9E10; Santa Cruz Biotechnology; dil. 1:1000), PARP (VIC 5; Boehringer Mannheim, Germany; dil. 1:2000), bcl-2 (clone 124; Dako SA, Glostrup, Denmark; dil. 1:200), bcl-$x_{L/S}$ (S-18; Santa Cruz Biotechnology; dil 1:500); Fas-L (N-20; Santa Cruz Biotechnology; dil. 1:1000), bax (N20; Santa Cruz Biotechnology; dil 1:500) were used. Peroxidase-labelled anti-mouse and anti-rabbit (Amersham Pharmacia Biotech; Milan, Italy, dil. 1:10 000) were used according to the manufacturer's instructions. To check the amount of proteins transferred to nitrocellulose membrane, heat shock protein (HSP) was used as control and detected by an anti HSP 72/73 mAb (Ab-1, clone W27, Calbiochem, Cambridge, MA, USA). The relative amount of the transferred proteins was quantified by scanning the autoradiographic films with a gel densitometer scanner (Bio-Rad) and normalized to the related HSP 72/73 amounts.

### CAT assay

NF-*k*B- and p53-mediated transcriptional activity was evaluated by CAT assay using the 4X(NF-*k*B)tkCAT and $PG_{13}$-CAT reporters respectively, as previously described (Biroccio et al, 1999; Ricca et al, 2000). To evaluate p53-mediated transcriptional activity, CAT assay was performed on MCF7 ADR cells 12 h after 5 μg ml$^{-1}$ cisplatin (DDP) or 15 μM ADR treatment, and on H460 cells 12 h after exposure to 1 μM ADR.

Transfection of cells was carried out by electroporation in the presence of 15 μg of CAT reporter genes and 4 μg of β-galactosidase (β-gal) expression vector PEQ176 to measure transfection efficiency. 48 h after transfection proteins were extracted by 3 cycles of rapid freezing and thawing. Protein concentration was quantified using the bicinchoninic acid protein assay reagent (BCA, Pierce, Chemical Co, Rockford, IL). CAT activity in equal numbers of β-gal units from different transfections was measured by using $^{14}$C-chloramphenicol and acetyl coenzyme A. Acetylated chloramphenicol was separated from non-acetylated chloramphenicol by thin-layer chromatography and quantified after autoradiography by means of slide scanner (Bio-Rad).

### Cytofluorimetric analysis

The percentage of cells in the different phases of cell cycle and in the sub-$G_1$ peak was estimated as previously described (Del Bufalo et al, 1996) using a CELLQuest software (Becton Dickinson, Heidelberg, Germany). For the analysis of reactive oxygen species (ROS) production, cells were collected, assayed for viability by trypan blu dye exclusion and then incubated for 45 min at 37˚C with 4 μM dihydroethidium (DHE, Molecular Probes, Eugene, OR, USA). Cytofluorimetric analysis was performed at the end of DHE staining.

### Terminal deoxynucleotide transferase (TdT)-mediated dUTP nick-end labelling (TUNEL)

The immunochemical detection of apoptotic cells was performed by TUNEL assay using the *in situ* cell death detection kit (Boehringer Mannheim), as previously reported (Leonetti et al,

© 2001 Cancer Research Campaign

1999). Briefly, 50 μl of TUNEL reaction mixture was applied to the cytospin preparation and the incubation performed at 37˚C for 60 min. The slides were then washed 3 times in PBS for 5 min, stained with Hoechst and examined by fluorescence microscope.

### Evaluation of tumorigenicity in athymic mice

6–8-week-old female CD-1 nude (nu/nu) mice, purchased from Charles River Laboratory (Calco, Italy) were used. All procedures involving animals and their care were described previously and were in accordance with national and international laws and policies (Leonetti et al, 1999). Each experimental group included 10 animals. To assess *in vivo* tumorigenicity we injected into the hind leg muscles of the mice different numbers of viable tumour cells (from $5 \times 10^5$ to $2 \times 10^7$). Mice were observed daily to establish the tumour take, the median time of tumour appearance and the tumour weights as reported previously (Leonetti et al, 1999). The results were analysed by the Mann–Whitney U test for statistical significance. Differences were considered significant at $P$ values < 0.05 (2-sided).

### RESULTS

### Generation of relA over-expressing clones

A mammalian expression vector for *relA* was used for generation of stable transfectants from MCF7 ADR line. 3 clonal cell lines stably over-expressing relA (AP2, AP3, AP14) and a APneo control clone, transfected with the vector carrying only the *neo*-resistance gene, were chosen for the experiments. Figure 1A shows Western blot analysis on nuclear extracts of relA/p65 protein levels in the MCF7 ADR line, APneo, AP2, AP3 and AP14 clones. Densitometric analysis normalized to HSP 72/73 expression reveals about 4–5-fold average increase of nuclear relA expression in all relA transfectants if compared to the levels observed in MCF7 ADR parental cells. The transfection with the control vector did not produce any change in relA nuclear levels. To investigate whether the relA over-expression was competent for transactivation. NF-*k*B-mediated transcriptional activity was evaluated. For this purpose CAT assay using a CAT reporter gene under the control of 4 copies of the IL-6 NF-*k*B-binding element was performed in the 3 relA transfectants, APneo clone and in parental cells. Figure 1B shows that the CAT activity, almost identical in the parental cell line and the APneo control clone, is up-regulated by relA over-expression in all the relA transfectants. Densitometric analysis revealed that the ratio between acetylated and unacetylated $^{14}$C-cloramphenicol (CAT activity), is about 6-fold higher in all relA clones compared to control cells. This result indicates that the stable increase of nuclear relA protein levels obtained in relA transfectants confers a corresponding increase in NF-*k*B-mediated transcriptional activity.

### Tumorigenicity of relA transfectants

To evaluate whether over-expression of relA might modify tumorigenicity of MCF7 ADR, different numbers of MCF7 ADR, APneo, AP2, AP3 and AP14 cells were injected intramuscularly into nude mice and tumour take and median time of tumour appearance were assessed (Table 1). For inoculum size ranging from $2 \times 10^7$ to $5 \times 10^5$

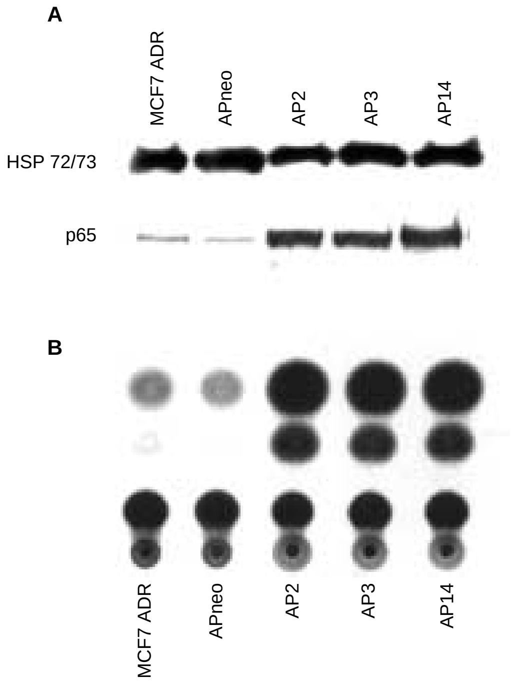

**Figure 1** (**A**) Western blot analysis of the p65 protein in the nuclear lysates of MCF7 ADR line, APneo control clone and 3 relA over-expressing clones (AP2, AP3, AP14). The relative amount of the transferred relA protein was quantified and normalized to the corresponding HSP 72/73 protein amount. (**B**) NF-*k*B-mediated transcriptional activity of MCF7 ADR line, APneo control clone and 3 relA over-expressing clones (AP2, AP3, AP14) evaluated by CAT assay

**Table 1** Tumorigenicity of MCF7 ADR, APneo control clone, and AP2, AP3, AP14 relA transfectants

| | No. of cells injected | Tumour take[a] (mice with tumours/mice injected) | Tumour latency[a] (days) |
|---|---|---|---|
| MCF7 ADR | $2 \times 10^7$ | 6/6 | 8 |
| | $1 \times 10^7$ | 6/6 | 10 |
| | $5 \times 10^6$ | 6/6 | 15 |
| | $1 \times 10^6$ | 6/6 | 20 |
| | $5 \times 10^5$ | 6/6 | 32 |
| APneo | $2 \times 10^7$ | 6/6 | 8 |
| | $1 \times 10^7$ | 6/6 | 10 |
| | $5 \times 10^6$ | 6/6 | 15 |
| | $1 \times 10^6$ | 6/6 | 20 |
| | $5 \times 10^5$ | 6/6 | 32 |
| AP2 | $2 \times 10^7$ | 4/6 | 38 |
| | $1 \times 10^7$ | 3/6 | 39 |
| | $5 \times 10^6$ | 1/6 | NA[b] |
| | $1 \times 10^6$ | 0/6 | NA |
| | $5 \times 10^5$ | 0/6 | NA |
| AP3 | $2 \times 10^7$ | 4/6 | 39 |
| | $1 \times 10^7$ | 3/7 | 43 |
| | $5 \times 10^6$ | 2/8 | NA |
| | $1 \times 10^6$ | 0/7 | NA |
| | $5 \times 10^5$ | 0/7 | NA |
| AP14 | $2 \times 10^7$ | 3/5 | 38 |
| | $1 \times 10^7$ | 2/5 | 43 |
| | $5 \times 10^6$ | 1/5 | NA |
| | $1 \times 10^6$ | 0/7 | NA |
| | $5 \times 10^5$ | 0/7 | NA |

[a]Tumour take and tumour latency were analysed until day 45 after cell injection. [b]NA = not assessable.

*© 2001 Cancer Research Campaign*

cells, parental and neo-transfected cells resulted in tumours in all the animals injected. Whereas, the injection of $2 \times 10^7$ relA over-expressing cells resulted in tumours in only 60–67% of animals. Tumour take of relA transfectants decreases to values of 40–50% and 17–25%, respectively, when $1 \times 10^7$ and $5 \times 10^6$ relA over-expressing cells were injected. 45 days after injection no tumours were observed in mice injected with $1 \times 10^6$ and $5 \times 10^5$ relA cells. The analysis of the median time of tumour appearance revealed that mice injected with $2 \times 10^7$ relA over-expressing cells began to develop tumours 38 days after injection, whereas the same number of parental and neo-transfected cells resulted in tumours after only 8 days ($P$ = 0.0095). Consequently, tumour volumes 45 days after injection of $2 \times 10^7$ relA over-expressing cells were significantly smaller (mean tumour weight = 157 ± 19 mg) than those obtained by injections of the same number of parental and neo-transfected cells (mean tumour weight = 663 ± 86 mg) with $P$ value of 0.0012.

### *In vitro* cell proliferation and cell cycle distribution of relA transfectants

Since relA and other members of the rel/NF-*k*B family have been demonstrated to affect proliferation of different model systems (Bash et al, 1997; Sheehy and Schlissel, 1999), we investigated the effects of the relA over-expression on cell growth and cell cycle distribution. Figure 2A shows the growth curves of the APneo control clone and 2 relA over-expressing clones. It appears evident that during the exponential phase of growth, relA over-expressing clones show a slight reduction in cell proliferation, when compared to the APneo clone. Moreover, a different saturation density characterized relA transfectants from neo-transfected cells. In fact, the mean cell number to reach the saturation density is $1.2 \times 10^6$ and $2.8 \times 10^6$ for relA transfectants and APneo clone, respectively. In addition, at day 7 of growth a decrease in cell number is observed in relA transfectants, while neo-transfected cells remain in the plateau phase of growth. Figure 2B illustrates APneo, AP2 and AP14 cell distribution in the different phases of the cell cycle. The analysis, performed from day 4 to 7 of growth curve, demonstrated an accumulation of relA overexpressing cells at the $G_1$ phase of cell cycle, already evident at day 4, with a concomitant decrease in the percentage of cells in S compartment. The relA over-expression did not affect the number of cells in the $G_2$/M phase. Results obtained from the analysis of the MCF7 ADR line and the AP3 clone were similar to those obtained, respectively, for APneo and AP14 clones (data not shown).

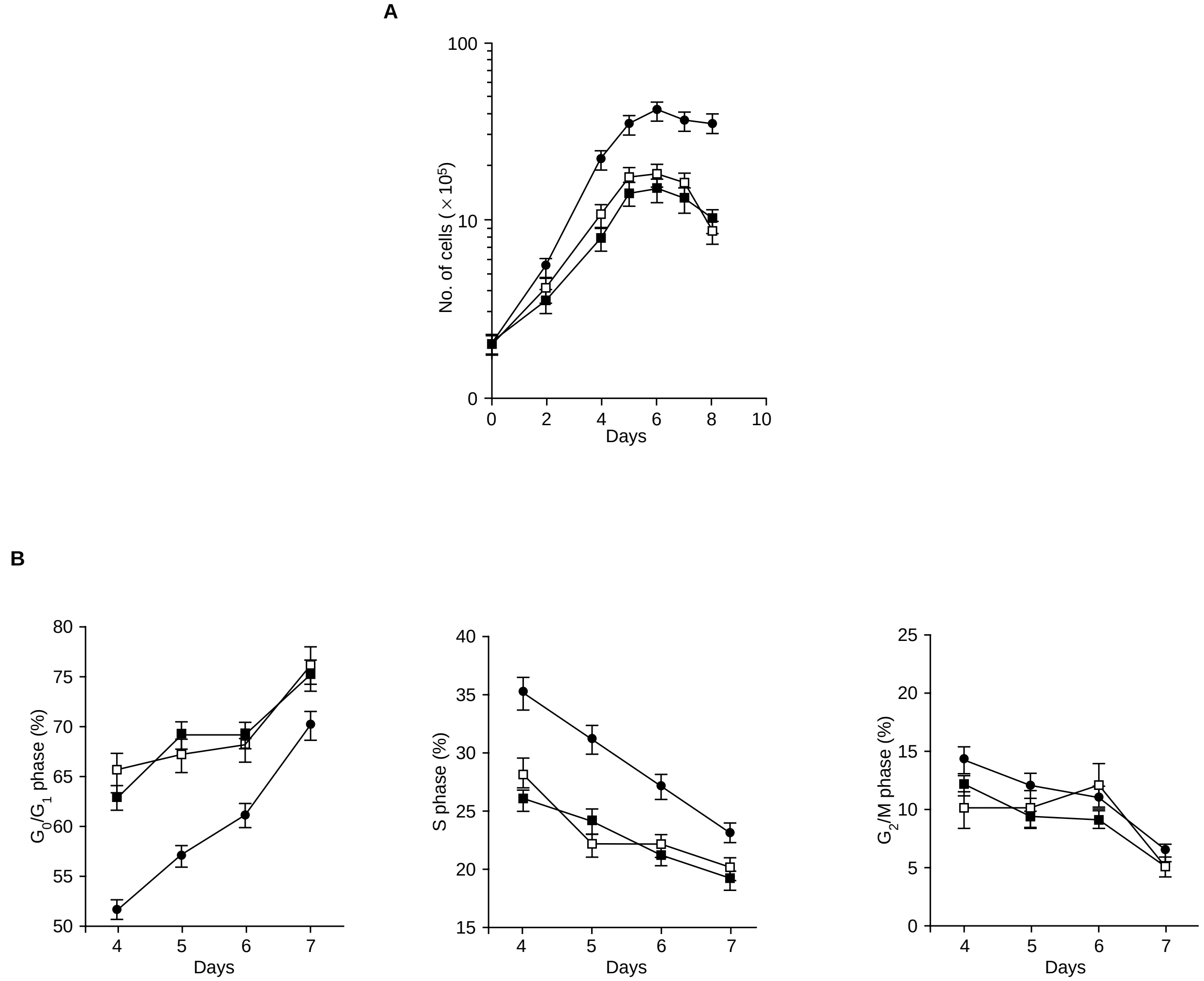

**Figure 2** *In vitro* cell proliferation (**A**) and cell cycle distribution (**B**) of APneo control clone (●), AP2 (■) and AP14 (□) relA transfectants. The data are the mean of 3 independent experiments with standard deviation

### Effect of relA over-expression on apoptotic cell death

Since NF-*k*B is involved in the regulation of apoptosis in several systems (Lin et al, 1995, 1999; Kaltschmidt et al, 2000), the reduction in cell number observed during cell growth and the decrease in tumorigenicity observed in relA transfectants led us to hypothesize that NF-*k*B activity could be involved in apoptosis induction. With this aim, we carried out a cytofluorimetric assessment of the DNA content, checking for the appearance of the sub-$G_1$ apoptotic population during the *in vitro* culture of control cells and relA transfectants. As reported in Figure 3A no cells with a DNA content lower than diploid appear from the analysis of APneo cells while a well-defined sub-$G_1$ population is evident in AP2 and AP14 clones at day 6 of the growth curve. The percentage of hypo-diploid cells is about 35% in both AP2 and AP14 clones. When the same analysis was performed at day 8 of cell culture the percentage of hypo-diploid cells was more than 80% for AP2 and AP14 cells, and only 10% for control cells (data not shown). TUNEL assay (Figure 3B) performed at day 6 of cell culture confirmed the cytofluorimetric data, demonstrating the presence of apoptotic cells both in AP2 and AP14 clones and the absence in APneo control cells. Results obtained from the analysis of the MCF7 ADR line and the AP3 clone were similar to those obtained, respectively, for APneo and AP14 clones (data not shown).

### Effect of relA over-expression on apoptosis-related protein expression

To identify molecular events which may account for the NF-*k*B-induced apoptosis described above, we examined whether the product of genes, involved in the regulation of the apoptotic process, such as c-myc, p53, bcl-$x_{L/S}$, bcl-2, bax, Fas-L, was modulated after relA over-expression. While we were unable to find significant changes in c-myc, bax, bcl-2, bcl-$x_L$ and Fas-L expression between control cells and relA transfectants, Western blot analysis of p53 protein revealed an increase of p53 protein levels of about 4–5-fold when relA over-expressing cells were compared to control cells (Figure 4). bcl-$x_S$ protein was not detected in our experimental conditions (data not shown).

The high level of p53 shown by Western blot analysis in the MCF7 ADR cells and the lack of activation of bax protein in relA transfectants expressing higher level of p53 than the parental line, led us to hypothesize that MCF7 ADR cells possess mutated p53 protein. Thus, the p53 transcriptional activity over a p53-specific promoter was evaluated by CAT assay after exposure to DDP and ADR, 2 genotoxic damage-inducing agents. Figure 5 shows that the activity of the $PG_{13}$-CAT reporter gene is undetectable both in untreated and DDP- or ADR-treated cells demonstrating that endogenous p53 was devoid of transcriptional activity.

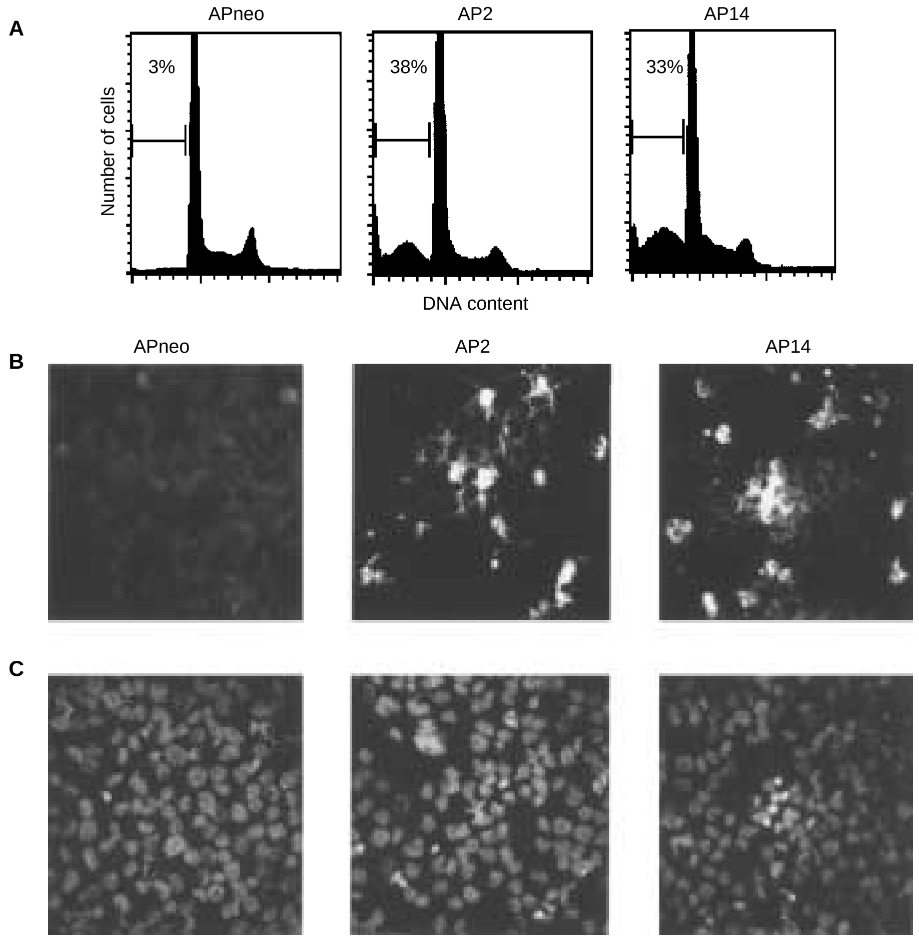

**Figure 3** (**A**) Cytofluorimetric analysis of DNA content in APneo control clone, and 2 relA over-expressing clones (AP2 and AP14). The percentage of cells with a sub-$G_1$ peak is reported. TUNEL assay (**B**) and Hoechst staining (**C**) of cytospin preparations from APneo control clone, and 2 relA over-expressing clones (AP2 and AP14). The analyses were performed at day 6 of growth curve

 *© 2001 Cancer Research Campaign*

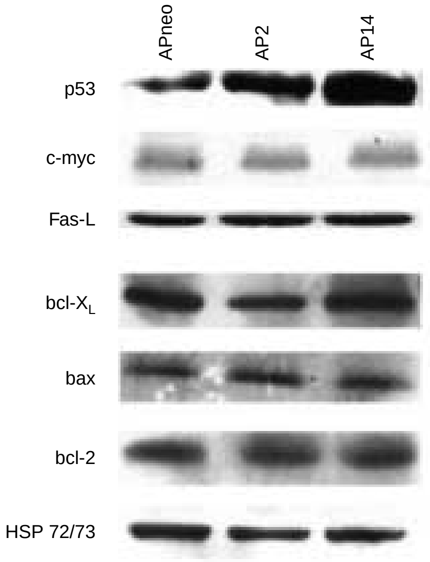

**Figure 4** Western blot analysis of p53, c-myc, Fas-L, bcl-x$_L$, bax and bcl-2 proteins in the cellular lysates of APneo control clone and 2 relA over-expressing clones (AP2 and AP14). The relative amounts of the transferred proteins were quantified and normalized to the correspondent HSP 72/73 protein amounts

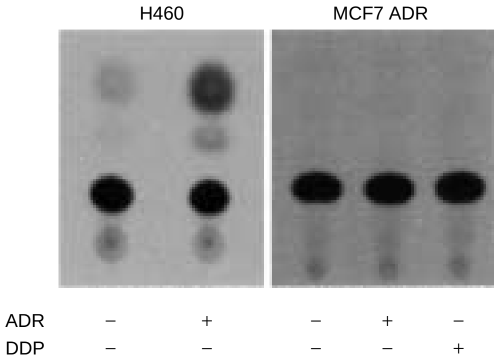

**Figure 5** Activity of the $PG_{13}$-CAT reporter in MCF7 ADR cells untreated or treated with 5 µg ml$^{-1}$ DDP or 15 µM ADR for 12 h. H460 cells treated with 1 µM ADR for 12 h were used as a positive control

### Effect of relA over-expression on ROS production

Since a strong correlation between NF-*k*B activation and ROS-induced apoptosis has recently been demonstrated (Dumont et al, 1999), we evaluated the intracellular content of ROS in control and relA over-expressing cells (Figure 6A). Gating the viable cells by means of forward- and side-scatter values assessment, no change in the relative fluorescence for APneo clone was observed at day 5 of the growth curve, while a clear increase in fluorescence is evidenced in AP2 and AP14 clones, the percent of cells with high-ROS content being 52% and 66%, respectively.

As a final biochemical event of apoptotic cell death, the cleavage of PARP substrate was evaluated. Figure 6B shows the results of Western blot analysis on total protein lysates from APneo, AP2 and AP14 clones. 116 kDa PARP was detected in lysates from both neo- and relA transfectants, while the 85 kDa cleavage product was only observed in lysates from the relA transfectants.

All the results obtained in AP2 and AP14 were similar to those observed in the AP3 clone (data not shown). No difference between the MCF7 ADR parental cells and the APneo clone were observed (data not shown).

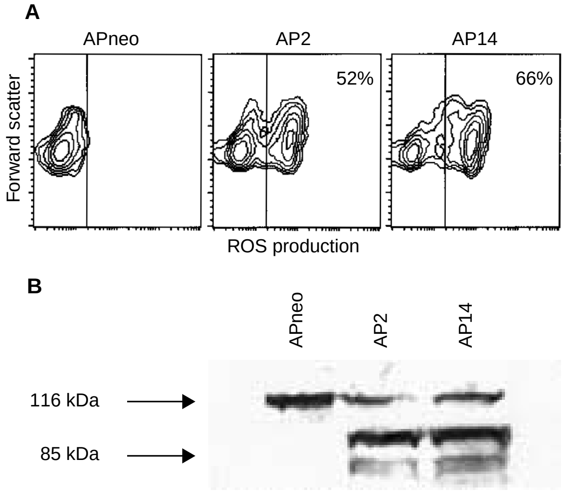

**Figure 6** Cytofluorimetric analysis of ROS (**A**), and Western blot analysis of PARP protein (**B**) in APneo control clone and 2 relA over-expressing clones (AP2 and AP14)

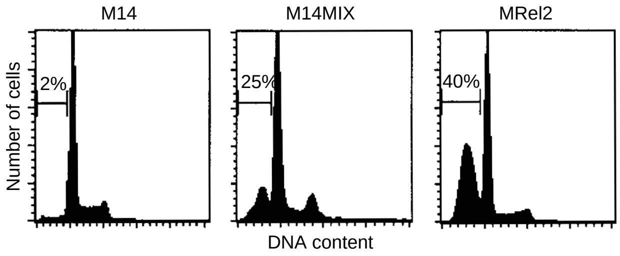

**Figure 7** Cytofluorimetric analysis of DNA content in M14 parental line, a relA over-expressing mixed population (M14MIX), and a relA over-expressing clone (MRel2). The percentage of cells with a sub-$G_1$ peak is reported

### Effect of relA over-expression on apoptotic cell death of melanoma cells

To examine whether NF-*k*B increased activity through relA over-expression results in the induction of apoptosis in another tumour cell line other than in MCF7 ADR model, we transfected the M14 human melanoma cell line with the same *relA* expression vector used for MCF7 ADR cell transfection. Parental cells, a relA over-expressing clone (MRel2) and a relA over-expressing mixed population (M14MIX) were studied to evaluate the induction of apoptosis by means of cytofluorimetric measure of DNA content. As shown in Figure 7 only relA over-expressing cells, either from the clonal or the mixed population, undergo apoptotic cell death as revealed from the clear appearance of the characteristic hypo-diploid peak. The share of hypo-diploid cells is 25% and 40% for M14MIX and MRel2 clone, respectively. These data confirmed and extended our previous observation obtained on MCF7 ADR cells, attesting for a more general phenomenon which links induced activation of NF-*k*B and apoptosis in human tumour cells.

### DISCUSSION

We recently demonstrated that the increased tumorigenic, metastatic and angiogenic potential induced by bcl-2 over-expression in the MCF7 ADR human breast cancer cell line, is accompanied by the

© 2001 Cancer Research Campaign enhancement of NF-*k*B transcriptional activity (Del Bufalo et al, 1997; Biroccio et al, 2000; Ricca et al, 2000).

In this paper we investigated the direct effects of an increased constitutive NF-*k*B activity on the malignant behaviour of MCF7 ADR cells by over-expressing the NF-*k*B subunit relA/p65.

Surprisingly, we found that a sustained activation of NF-*k*B leads to a great reduction of the MCF7 ADR tumorigenic potential in nude mice. In fact, the injection of $5 \times 10^5$ parental cells results in tumours in all the animals, while no animals developed tumours when injected with the same number of relA over-expressing cells. In addition, $2 \times 10^7$ control cells give rise to tumours in a very short time (8 days), while the same number of relA transfected cells gives rise to tumours in only 4/6 mice and the tumour latency was more then 4 times longer than that of MCF7 ADR tumours. The decrease in tumorigenicity was accompanied by a reduction of *in vitro* cell proliferation. Our data are in agreement with those of other authors demonstrating that elevated levels of relA induced a $G_1$ cell cycle arrest (Sheehy and Schlisse, 1999). Also the over-expression of other members of the rel/NF-*k*B, such as c-rel has been found to induce growth arrest at the $G_1$/S-phase transition (Bash et al, 1997). Finally, studies performed on murine and human epidermis transgenic for proteins activating or inhibiting NF-*k*B function, demonstrated that NF-*k*B function is important for cellular growth inhibition in stratified epithelium (Seitz et al, 1998).

We also found that the decreased tumorigenicity was associated with activation of the apoptotic programme. The ability of NF-*k*B to induce apoptosis was also observed in M14 human melanoma cell line.

To address the molecular and biochemical events responsible for the NF-*k*B-induced apoptosis, we investigated the involvement of some apoptosis-modulator proteins. No difference between parental cells and relA transfectants was found in the expressions of Fas-L, bcl-2, bcl-$x_L$, bax and c-myc proteins, thus, indicating these proteins were not involved in NF-*k*B-induced apoptosis. On the contrary, the p53 protein, whose gene is known to be a target of NF-*k*B transcriptional activity (Wu and Lozano, 1994), was found to be increased after relA over-expression. However, we have evidence that p53 is devoid of transcriptional activity, indicating that the mechanisms responsible of p53 inactivation are able to block also high level of p53. Thus, even though mutant p53 gain of function has been demonstrated (Blandino et al, 1999), NF-*k*B-mediated apoptosis in MCF7 ADR cells is probably independent of this protein. The inability of p53 to increase bax levels in relA transfectants and our previous results, demonstrating that p53 is segregated in the cytoplasm in spite of the wild-type configuration (Del Bufalo et al, 1996), support the evidence that p53 is in an inactivated form.

Our data provide evidence that the massive apoptotic cell death induced by relA overexpression is associated to ROS generation. In fact, a significant percentage of ROS production is exclusively observed in the relA transfectants. This is in agreement with other findings demonstrating that ROS *per se* are potent inducers of apoptosis (Kroemer et al, 1997) and that the hydrogen peroxide-induced apoptosis, requires the release of mitochondria-derived ROS and the activation of NF-*k*B (Dumont et al, 1999).

In addition, our results, demonstrating the ability of p65 over-expression to induce apoptosis, are in agreement with published data evidencing NF-*k*B implication in inducing cell death in certain cells such as neurons, Schwann cells, prostate carcinoma cells, and embryonic kidney cells (Lin et al, 1995; Carter et al, 1996; Grilli et al, 1996; Grimm et al, 1996; Lin et al, 1999). As suggested by Arlt, the fact that NF-*k*B activation triggers apoptosis might involve particular NF-*k*B target genes that are of functional duality in terms of growth control and survival and that are subject to modulation by many other cellular signals (Arlt et al, 2001). Moreover, even though some papers evidenced the NF-*k*B ability to protect against instead of triggering apoptosis, the majority of these results demonstrated the anti-apoptotic role of NF-*k*B in response to the induction of apoptosis by different stimuli (Carter et al, 1996; Grilli et al, 1996; Grimm et al, 1996; Kaltschmidt et al, 2000). On the contrary, in our model we evaluated the effect of NF-*k*B over-expression in the absence of stimuli inducing apoptosis. Moreover, even though a few studies have demonstrated reduced cell growth, adhesion and *in vivo* tumour growth by antisense inhibition of the p65 subunit (Kitajima et al, 1992; Higgins et al, 1993; Khaled et al, 1996), some authors disputed the specificity of these effects (Andela et al, 2000), and other authors found the lack of an effect of NF-*k*B suppression on *in vivo* tumour growth (Khaled et al, 1996).

The data presented in this paper appear to be in contrast to that previously published by our group where bcl-2 over-expression in MCF7 ADR cells enhances NF-*k*B activity and increases the tumorigenic, metastatic and angiogenic potential (Del Bufalo et al, 1997; Biroccio et al, 2000; Ricca et al, 2000). We suggest that in the complex onset and progression of human cancers, amplification and over-expression of cellular rel/NF-*k*B genes are likely to provide the necessary threshold for nuclear rel/NF-*k*B activity. As the enhancement in NF-*k*B activity results in activation of the apoptotic programme and on the basis of the data presented here, we can speculate that MCF7 ADR cells over-expressing bcl-2 balances the pro-apoptotic NF-*k*B property with the anti-apoptotic activity of bcl-2. In addition, we have evidence that *bcl-2* acts, through modulation of other factors (Del Bufalo et al, 1997), other than through NF-*k*B activation (Ricca et al, 2000), thus, suggesting that *bcl-2* might modulate the transcription activity of other factors that are responsible for the phenotype observed after bcl-2 over-expression. As such, the increase of NF-*k*B activity following bcl-2 or p65 over-expression may represent 2 different stimuli for MCF7 ADR cells, the first increasing tumorigenicity and the second one inducing apoptosis and reducing tumorigenicity. It is also possible that the levels of NF-*k*B activity play a role in this phenomenon. In fact, the indirect increase in NF-*k*B transcriptional activity induced by bcl-2 overexpression (about 3 times greater in bcl-2 transfectants than the parental line) corresponds to lower levels of those obtained directly increasing NF-*k*B activity by transfection (about 6 times greater in bcl-2 transfectants than the parental line). As suggested by Gilmore, partial induction of nuclear NF-*k*B activity may be protective, while full induction of NF-*k*B activity may itself be toxic or may induce apoptosis (Gilmore, 1997). Thus, over-induction of nuclear NF-*k*B activity may be as lethal to tumour cells as inhibition of this activity.

In conclusion, the data presented in this paper add new insights to the complex linkage between the NF-*k*B activity and the regulation of the apoptotic process and tumorigenic potential. However, it is clear that there is no general role for NF-*k*B in the regulation of apoptosis and tumorigenicity but rather NF-*k*B functions as a complex cell-specific and stimuli-specific regulator of this phenomenon. Any use of effective interventions against cancer, targeting NF-*k*B signalling will rely on a full understanding of the role of cell type, NF-*k*B subunit composition, target gene functions and promoter sensitivity to NF-*k*B.

 *© 2001 Cancer Research Campaign*

### ACKNOWLEDGEMENTS

We are grateful to Mrs Simona Righi for typing this manuscript and to Paula Franke for language revision. Dr Alfredo Ricca is the recipient of a fellowship from the Italian Foundation for Cancer (FIRC). This work was supported by Italian Association for Cancer Research and Ministero della Sanità.

### REFERENCES

Abbadie C, Kabrun N, Bouali F, Smardova J, Stéhelin D, Vandenbunder B and Enrietto PJ (1993) High levels of c-rel expression are associated with programmed cell death in the developing avian embryo and in bone marrow cells *in vitro*. *Cell* **75**: 899–912

Andela VB, Schwarz EM, Puzas JE, O'Keefe RJ and Rosier RN (2000) Tumor metastasis and the reciprocal regulation of prometastatic and antimetastatic factors by nuclear factor κB. *Cancer Res* **60**: 6557–6562

Arlt A, Grobe O, Sieke A, Kruse M-L, Fölsch UR, Schmidt WE and Schäfer H (2001) Expression of the NF-*k*B target gene IEX-1 (p22/PRG1) does not prevent cell death but instead triggers apoptosis in Hela cells. *Oncogene* **20**: 69–76

Baeuerle P and Henkel T (1994) Function and activation of NF-*k*B in the immune system. *Annu Rev Immunol* **12**: 141–179

Baeuerle PA and Baltimore D (1996) NF-kappa B: ten years after. *Cell* **87**: 13–20

Bargou RC, Emmerich F, Krappmann D, Bommert K, Mapara MY, Arnold W, Royer HD, Grinstein E, Greiner A, Scheidereit C and Dörken B (1997) Constitutive nuclear factor-κB-relA activation is required for proliferation and survival of Hodgkin's disease tumor cells. *J Clin Invest* **100**: 2961–2969

Bash J, Zong W-X and Gélinas C (1997) c-rel arrests the proliferation of HeLa cells and affects critical regulators of the $G_1$/S-phase transition. *Mol Cell Biol* **17**: 6526–6536

Biroccio A, Del Bufalo D, Ricca A, D'Angelo C, D'Orazi G, Sacchi A, Soddu S and Zupi G (1999) Increase of BCNU sensitivity by wt-p53 gene therapy in glioblastoma lines depends on the administration schedule. *Gene Therapy* **6**: 1064–1072

Biroccio A, Candiloro A, Mottolese M, Sapora O, Albini A, Zupi G and Del Bufalo D (2000) Bcl-2 overexpression and hypoxia synergistically act to modulate vascular endothelial growth factor expression and in vivo angiogenesis in a breast carcinoma line. *Faseb J* **14**: 652–660

Blandino G, Levine AJ and Oren M (1999) Mutant p53 gain of function: differential effects of different p53 mutants on resistance of cultured cells to chemotherapy. *Oncogene* **18**: 477–485

Carter BD, Kaltschmidt C, Kaltschmidt B, Offenhauser N, Bohm Matthaei R, Baeuerle PA and Barde YA (1996) Selective activation of NF-kappaB by nerve growth factor through the neurotrophin receptor p75. *Science* **272**: 542–545

Del Bufalo D, Biroccio A, Soddu S, Laudonio N, D'Angelo C, Sacchi A and Zupi G (1996) Lonidamine induces apoptosis in drug-resistant cells independently of the p53 gene. *J Clin Invest* **98**: 1165–1173

Del Bufalo D, Biroccio A, Leonetti C and Zupi G (1997) Bcl-2 overexpression enhances the metastatic potential of a human breast cancer line. *Faseb J* **11**: 947–953

Dumont A, Hehner SP, Hofmann TG, Ueffing M, Dröge W and Schmitz ML (1999) Hydrogen peroxide-induced apoptosis is CD95-independent, requires the release of mitochondria-derived reactive oxygen species and the activation of NF-*k*B. *Oncogene* **18**: 747–757

Gilmore TD (1997) Clinically relevant findings. *J Clin Invest* **100**: 2935–2936

Grilli M and Memo M (1999) Nuclear factor kappa B/Rel proteins: a point of convergence of signaling pathways relevant in neuronal function and dysfunction. *Biochem Pharmacol* **57**: 1–7

Grilli M, Pizzi M, Memorandum M and Spano P (1996) Neuroprotection by aspirin and sodium salicylate through blockade of NF-kappaB activation. *Science* **274**: 1383–1385

Grimm S, Bauer MK, Baeuerle PA and Schulze Osthoff K (1996) Bcl-2 down-regulates the activity of transcription factor NF-kappaB induced upon apoptosis. *J Cell Biol* **134**: 13–23

Higgins KA, Perez JR, Coleman TA, Dorshkind K, McComas WA, Sarmiento UM, Rosen CA and Narayanan R (1993) Antisense inhibition of the p65 subunit of NF-*k*B blocks tumorigenicity and causes tumour regression. *Proc Natl Acad Sci USA* **90**: 9901–9905

Huang S, DeGuzman A, Bucana CD and Fidler IJ (2000) Nuclear factor-κB activity correlAtes with growth, angiogenesis, and metastasis of human melanoma cells in nude mice. *Clinical Cancer Res* **6**: 2573–2581

Kaltschmidt B, Kaltschmidt C, Hofmann TG, Hehner SP, Dröge W and Schmitz ML (2000) The pro- or anti apoptotic function of NF-*k*B is determined be the nature of the apoptotic stimulus. *Eur J Biochem* **267**: 3828–3835

Khaled Z, Benimetskaya L, Zeltser R, Khan T, Sharma HW, Narayanan R and Stein CA (1996) Multiple mechanisms may contribute to the cellular anti-adhesive effects of phosphorothioate oligodeoxynucleotides. *Nucleic Acid Res* **24**: 737–745

Kitajima I, Shinohara T, Bilakovics J, Brown DA, Xu X and Nerenberg M (1992) Ablation of transplanted HTLV-I Tax-transformed tumors in mice by antisense inhibition of NF-kappa B *Science* **258**: 1792–1795

Krappmann D, Emmerich F, Kordes U, Scharschmidt E, Dörken B and Scheidereit C (1999) Molecular mechanisms of constitutive NF-*k*B/Rel activation in Hodgkin/Reed-Sternberg cells. *Oncogene* **18**: 943–953

Kroemer G, Zamzami N and Susin SA (1997) Mitochondrial control of apoptosis. Immun. *Today* **18**: 45–51

Leonetti C, Biroccio A, Candiloro A, Citro G, Fornari C, Mottolese M, Del Bufalo D and Zupi G (1999) Increase of cisplatin sensitivity by c-myc antisense ologodeoxynucleotides in a human metastatic melanoma inherently resistant to cisplatin. *Clinical Cancer Res* **5**: 2588–2595

Lin B, Williams-Skipp C, Tao Y, Schleicher MS, Cano LL, Duke RC and Scheinman RI (1999) NF-*k*B functions as both a proapoptotic and antiapoptotic regulatory factor within a single cell type. *Cell Death Diff* **6**: 570–582

Lin K-I, Lee S-H, Narayanan R, Baraban JM, Hardwick JM and Ratan RR (1995) Thiol agents and Bcl-2 identify an alphavirus-induced apoptotic pathway that requires activation of the transcription factor NF-*k*B. *J Cell Biol* **131**: 1149–1161

Nakshatri H, Bhat-Nakshatri P, Martin DA, Goulet RJ Jr., and Sledge GW Jr. (1997) Constitutive activation of NK-κB during progression of breast cancer to hormone-independent growth. *Mol Cell Biol* **17**: 3629–3639

Pahl HL (1999) Activators and target genes of Rel/NF-*k*B transcription factors. *Oncogene* **18**: 6853–6866

Polyak K, Xia Y, Zweier JL, Kinzler KW and Vogelstein B (1997) A model for p53-induced apoptosis. *Nature* **389**: 300–305

Rayet B and Gélinas C (1999) Aberrant rel/*nfkb* genes and activity in human cancer. *Oncogene* **18**: 6938–6947

Ricca A, Biroccio A, Del Bufalo D, Mackay AR, Santoni A and Cippitelli M (2000) Bcl-2 overexpression enhances NF-*k*B activity and induces MMP-9 transcription in human $MCF7^{ADR}$ breast cancer cells. *Int J Cancer* **86**: 188–196

Seitz CS, Lin Q, Deng H and Khavari PA (1998) Alterations in NF-*k*B function in transgenic epithelial tissue demonstrate a growth inhibitory role for NF-*k*B. *Proc Natl Acad Sci USA* **95**: 2307–2312

Sheehy AM and Schlissel MS (1999) Overexpression of relA causes $G_1$ arrest and apoptosis in a pro-B cell line. *J Biol Chem* **274**: 8708–8716

Sovak MA, Bellas RE, Kim DW, Zanieski GJ, Rogers AE, Traish AM and Sonenshein GE (1997) Aberrant nuclear factor-κ/Rel expression and the pathogenesis of breast cancer. *J Clin Invest* **100**: 2952–2960

Thanos D and Maniatis T (1999) NF-*k*B: a lesson in family values. *Cell* **80**: 529–532

Visconti R, Cerutti J, Battista S, Fedele M, Trapasso F, Zeki K, Miano MP, de Nigris F, Casalino L, Curcio F, Santoro M and Fusco A (1997) Expression of the neoplastic phenotype by human thyroid carcinoma cell lines requires NF-*k*B p65 protein expression. *Oncogene* **15**: 1987–1994

Wang CY, Mayo MW, Korneluk RG, Goeddel DV and Baldwin AS Jr. (1998) NF-kappaB antiapoptosis: induction of TRAF1 and TRAF2 and c-IAP1 and c-IAP2 to suppress caspase-8 activation. *Science* **281**: 1680–1683

Wu H and Lozano G (1994) NF-kappaB activation of p53. A potential mechanism for suppressing cell growth in response to stress. *J Biol Chem* **269**: 20067–20074

Wu MX, Ao Z, Prasad KV, Wu R and Schlossman SF (1998) IEX-1L, an apoptosis inhibitor involved in NF-kappaB-mediated cell survival. *Science* **281**: 998–1001

© 2001 Cancer Research Campaign 
